# Retraction: Podophyllotoxin and Rutin Modulate M1 (iNOS+) Macrophages and Mitigate Lethal Radiation (LR) Induced Inflammatory Responses in Mice

**DOI:** 10.3389/fimmu.2022.899504

**Published:** 2022-03-30

**Authors:** Frontiers Editorial Office

The journal retracts the 12 February 2019 article cited above.

Following publication, concerns were raised regarding the integrity of the data in the published figures. The authors failed to provide a satisfactory explanation during the investigation, which was conducted in accordance with Frontiers’ policies.

This retraction was approved by the Chief Editors of Frontiers in Immunology and the Chief Executive Editor of Frontiers. The authors do not agree to this retraction.

